# Cellular Origin of Spontaneous Ganglion Cell Spike Activity in Animal Models of Retinitis Pigmentosa

**DOI:** 10.1155/2011/507037

**Published:** 2010-09-29

**Authors:** David J. Margolis, Peter B. Detwiler

**Affiliations:** ^1^Program in Neurobiology and Behavior, Department of Physiology and Biophysics, University of Washington, Seattle, WA 98195, USA; ^2^Department of Neurophysiology, Brain Research Institute, University of Zurich, 8057 Zurich, Switzerland

## Abstract

Here we review evidence that loss of photoreceptors due to degenerative retinal disease causes an increase in the rate of spontaneous ganglion spike discharge. Information about persistent spike activity is important since it is expected to add noise to the communication between the eye and the brain and thus impact the design and effective use of retinal prosthetics for restoring visual function in patients blinded by disease. Patch-clamp recordings from identified types of ON and OFF retinal ganglion cells in the adult (36–210 d old) *rd1* mouse show that the ongoing oscillatory spike activity in both cell types is driven by strong rhythmic synaptic input from presynaptic neurons that is blocked by CNQX. The recurrent synaptic activity may arise in a negative feedback loop between a bipolar cell and an amacrine cell that exhibits resonant behavior and oscillations in membrane potential when the normal balance between excitation and inhibition is disrupted by the absence of photoreceptor input.

## 1. Introduction

Retinitis pigmentosa (RP) refers to a number of related diseases that result in the death of rod and cone photoreceptors causing blindness in about one in 3,500 people, nearly 2 million people worldwide. Not surprisingly, PubMed lists more than 7,000 papers on RP that provide an abundant source of information about the genetic, biochemical, physiological, and therapeutic characteristics of the disease. The goal of much recent work on RP has been to develop methods to restore vision by resuscitating the retina using gene therapy to repair the mutation that gives rise to the dystrophy [1] or by driving it artificially using neural prosthetics that are based on either electrical stimulation via implanted retinal electrodes [2] or optical stimulation via light activation of ectopically expressed photosensitive proteins [3–9]. The success of any of these approaches ultimately depends on the functional integrity of retinal ganglion cells (RGCs), the output cells of the retina whose axons carry spike-encoded information to the visual centers in the central nervous system. To make optimal use of ganglion cells for communicating with the brain, it is necessary to know how they are affected by the degenerative loss of photoreceptors and the accompanying changes in the cellular architecture of the retina [10–15].

## 2. RP Increases Spontaneous Spike Activity in Ganglion Cells

Out of the several thousand publications on RP, less than a dozen have addressed questions about the effects of retinal degeneration on RGC firing properties. The responses of individual cells cannot be evaluated using the electroretinogram (ERG), which is the widely employed standard method for assessing the functional changes in the retina resulting from loss of photoreceptor input. An early study by Drager and Hubel [16] based on extracellular single unit recordings from either optic nerve, superior colliculus (SC), or visual cortex reports an increase in spontaneous spike activity with maintained rhythmic firing in *rd1* mice that was not present in normal animals. The patterned spike activity was reversibly abolished by temporarily occluding blood flow to the eye, providing evidence of its retinal origin. The frequency of the persistent discharge was dependent on the anesthetic and ranged between 9–14 Hz. These findings were confirmed subsequently using autocorrelgrams to demonstrate the rhythmicity of maintained spike activity in units recorded from the SC in dystrophic but not nondystrophic Royal College of Surgeons (RCS) rats [17]. There are also reports of increased *c-fos-like* immunoreactivity in the superior colliculus and lateral geniculate nucleus in *rd1* mice and RCS rats that is eliminated by intraocular injection of TTX or optic nerve transection [18, 19]. The increase in *c-fos* expression was attributed to the generation of rhythmic input from retinal ganglion cells. 

The changes in RGC spike activity during the progression of photoreceptor degeneration has been documented more directly using extracellular single RGC recording in the RCS rat [20] as well as multielectrode array recordings in retina from the *rd1* mouse [21] and the P23H rat, an animal model of human autosomal dominant RP [22]. In agreement with the earlier accounts the single cell and multielectrode recordings showed a marked increase in the frequency of maintained spontaneous spike activity with rhythmic bursts [9, 21] in adult animals that have lost their ability to respond to light. An increase in glutamate-mediated excitatory signaling has also been observed in rodent models of RP using organic cations and immunoreactivity to map neuronal activity [11]. Taken together, the overall conclusion of these studies is that photoreceptor death due to degenerative disease leads to hyperactivity in ganglion cells.

It is important to understand the properties of the ongoing spike activity that is present in RP because it represents an undesirable noise source that degrades the communication between the eye and the brain that the aforementioned strategies to restore vision in patients blinded by degenerative disease depend upon. Here we review experiments designed to investigate the cellular mechanisms responsible for the increase in maintained spike activity and explore the retinal circuitry that may give rise to it.

## 3. RP-Induced Changes in Spike Activity in Identified Retinal Ganglion Cells

To determine whether RGC hyperactivity was caused by changes in the intrinsic properties of RGCs, such as ion channel function or distribution, or by altered synaptic input, intracellular recording was used to study the effect of photoreceptor loss on the electrophysiological properties of selected types of ganglion cells in *rd1* retina [23]. RGCs with the soma diameters (≥20 *μ*m)—which, by virtue of their large size, are referred to here as alpha cells [24]—were targeted for whole cell current or voltage clamp recording and filled by internal dialysis with an intracellular fluorescent indicator. Images obtained by 2-photon laser scanning fluorescent microscopy [25] were used to classify recorded RGCs as either ON, OFF transient, or OFF-sustained alpha cells, based on their dendrite stratification depth in the inner plexiform layer [24]. The use of morphological criteria to reliably identify RGC subtypes in blind animals is made possible by the fact that the dendritic morphology of ganglion cells is not affected by photoreceptor degeneration [23, 26].

**Figure 1:**
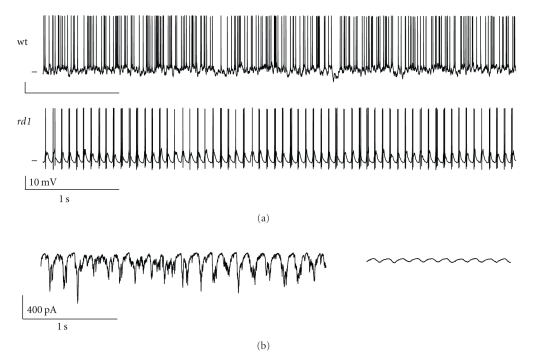
Spontaneous activity in *rd1* alpha ganglion cell. (a) Whole-cell current clamp recordings of ongoing spiking activity in wild-type (top) and *rd1* (bottom) ON-type retinal ganglion cells. Horizontal tick mark at left indicates −60 mV for wt and −70 mV for rd1 cells, respectively. (b) Whole-cell voltage clamp recording of ongoing synaptic currents in an *rd1* ON ganglion cell before (left) and after (right) bath application of CNQX.

Unlike ganglion cells from normal animals, which generate resting spike activity with no obvious temporal periodicity, the rate of spontaneous spike discharge in alpha RGCs from animals blinded by degeneration is increased and consists of continuous rhythmic bursts of spikes (Figure 1) with a beat frequency of ~10 Hz; the same frequency as the persistent discharge was reported by Drager and Hubel [16]. The clockwork firing of the alpha RGCs is maintained 24/7 in adult animals ranging in age from 36 to 210 days; experiments were not done on older animals. During this time the intrinsic network and electrophysiological properties of the cells were remarkably stable [23]. More specifically *rd1* alpha RGS retained the characteristic differences in the weights of excitatory and inhibitory synaptic inputs that ON and OFF cell types receive. They also continued to generate rebound excitation in OFF cells and gave rise to voltage-evoked dendrite calcium signals that were similar to those recorded from the dendrites of RGCs in non-dystrophic retina [27]. The rhythmic bursts of spikes that are a hallmark of *rd1* alpha RGC activity are triggered by oscillatory synaptic inputs as shown by the fact that they persist under voltage clamp recording conditions and are eliminated by CNQX, a glutamatergic blocker (Figure 1).

## 4. Source of Enhanced Synaptic Inputs

The presynaptic source of the synaptic inputs that give rise to rhythmic firing is not known. That *rd1* ON and OFF RGCs retain their normal distinguishing differences in the strengths of the excitatory and inhibitory inputs they receive, in spite of the ongoing oscillations in maintained synaptic activation, suggests that the organization and distribution of RGC contacts with presynaptic neurons have not been remodeled. The extensive changes in retina morphology that have been reported in this and other models of RP [28, 29] emerge in animals that are more than twice as old as the oldest animals used by Margolis [23]. While the slow onset of retinal remodeling makes it clearly important to document the accompanying changes in the cellular physiology of identified retinal neurons in older (P500) animals, this has not been done for purely practical reasons having to do with the required investments of time (nearly two years) and money (cost of maintaining a geriatric mouse colony). Hence the following discussion pertains to P36 to P210 *rd1* animals where it appears that functional changes have occurred but massive remodeling of the inner retina has not taken place.

**Figure 2:**
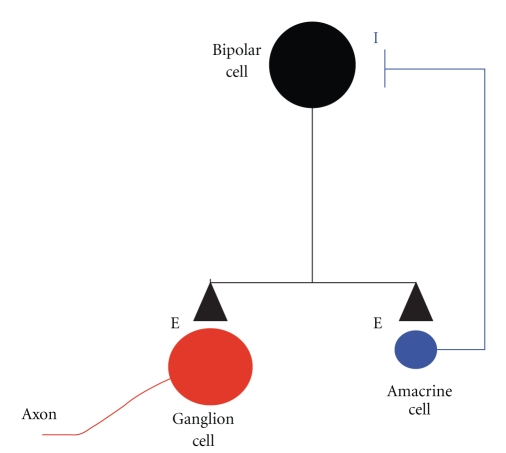
Retinal circuit that may give rise to spontaneous ganglion cell spike activity. The membrane potential of the amacrine cell oscillates spontaneously due to resonance (see text) which drives oscillatory release of inhibitory transmitter on to the bipolar cell causing oscillations in bipolar voltage triggering pulsatile release of excitatory transmitter on to the ganglion cell, causing rhythmic spike discharge, and the amacrine cell with negative feedback to the bipolar. The reverberating input to the ganglion cell arises from the presence of a negative feedback loop that includes a resonant oscillator.

Single cell recordings from bipolar cells isolated from dissociated *rd1* retina show no evidence of having intrinsic pacemaker activity that gives rise to spontaneous fluctuations in membrane potential [30]. This indicates that the rhythmic synaptic input to RGCs does not originate in bipolar cells suggesting instead that it first arises in a subset of amacrine cells. The underlying circuitry must, however, also include bipolar cells, since amacrine cell synaptic output is inhibitory and mediated by release of either GABA or glycine while the rhythmic synaptic input that drives ganglion hyperactivity is blocked by CNQX and is thus glutamatergic (Figure 1). A retinal circuit (Figure 2) that could give rise to the observed rhythmic spike discharge in ON and OFF RGCs begins with an amacrine cell having the necessary intrinsic combination of ion conductances to produce resonant oscillations in membrane voltage [31]. This is not an unusual property to ascribe to a member of the amacrine cell population where spontaneous oscillations in current and voltage have been reported to occur in starburst [32], wide field [33] and dopaminergic [34, 35] amacrines. In this speculative circuit, subthreshold oscillations in amacrine cell voltage, with or without amplification by voltage-gated conductances, are postulated to trigger oscillatory changes in inhibitory transmitter release, which, in turn, generate oscillations in the membrane potential of the bipolar cells they are synaptically coupled to. The resulting periodic variation in bipolar cell potential gives rise to pulsatile glutamate release and rhythmic excitatory synaptic input to RGCs. 

This hypothesized mechanism for oscillatory spike discharge could be tested by recording from bipolar cells in an intact dystrophic retina to determine if rhythmic changes in membrane voltage are present and sensitive to inhibitory synaptic blockade. Note that the proposed circuit cannot be rejected solely on the basis of finding no evidence of periodic fluctuations in baseline voltage in recordings from bipolar cells in retinal slices [36]. In such a preparation, there are affiliated uncertainties about whether the cellular connections required for rhythmic synaptic interactions have been disrupted in the process of slicing the retina. If the proposed feedback circuitry drives rhythmic spike activity in the *rd1* retina, it might be expected that oscillations in spike discharge would not be confined to local spatial areas, but would instead be rather widespread. Stasheff [21], however, did not find evidence of correlations in spiking between pairs of ganglion cells. This either suggests that ganglion cells are in fact independent, or that correlations exist but only on a spatial scale smaller than the 200 *μ*m spacing of the electrode array that the study made use of.

## 5. Oscillations Arising from Resonance in a Feedback Loop

**Figure 3:**
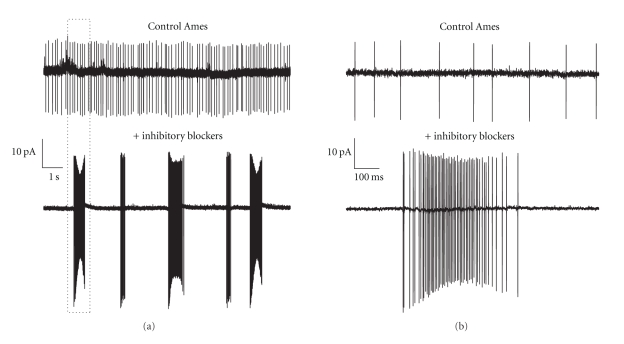
Loose patch extracellular recording of spontaneous spike activity from wild-type (non-dystrophic) retinal ganglion cell perfused with Control Ames solution without (top traces (a) and (b)) and with (lower traces (a) and (b)) the addition of mixture of inhibitory synaptic blockers containing 40 uM Gabazine, 50 uM TPMPA, and 1 uM Strychnine. Boxed region outlined by the dash lines in (a) are shown on a faster time in (b). The trace shown on an expanded time scale in control Ames (b) was taken from a region of trace in (a) that was shifted to the right to avoid the period of baseline instability.

In the proposed circuit the oscillations that give rise to rhythmic RGC spike discharge originate in an unidentified amacrine cell as a result of photoreceptor death and deafferentation. In this scenario, it is the loss of photoreceptor synaptic input that unbalances the circuitry of the normal retina and in so doing exposes the resonant membrane properties of an amacrine cell that is normally held in check in the functionally intact retina. Resonance is a consequence of the interactions between the active and passive membrane properties of a cell [24] that effectively combines a high-pass filter, arising from the presence of an active, that is, voltage-dependent, conductance [37], and a low pass filter that is an inherent consequence of the cell's passive membrane properties. The interplay between the two filters produces the equivalent of a notch filter that passes inputs with a select frequency band and rejects inputs with frequencies outside its band-pass. Changes in the input to the cell may influence the expression of resonant behavior and the generation of oscillations in two ways by changing the active and passive membrane properties that set the resonant frequency and by shifting the frequency of the input relative to the band-pass of the resonant filter, which under the right conditions can generate reverberating activity in a negative feedback loop. As anyone who has attempted to build an electronic feedback amplifier knows, the output of a circuit like the one we have proposed is much more likely to be oscillatory than stationary. Similarly, the output of a neural network, with multiple synaptic feedback loops, such as the retina, is particularly prone to oscillations. This notion is supported by recent results in non-dystrophic mouse retina showing that in the presence of a mixture of inhibitory synaptic blockers RGCs generate spontaneous bursts of spikes (Figure 3) that are eliminated by addition of CNQX, showing that the periodic bursts of activity are produced by excitatory synaptic input (Newkirk and Detwiler unpublished observations). These observations suggest that the synaptic circuitry in the healthy retina is critically tuned to establish a balance between excitation and inhibition in a way that minimizes resonance and optimizes the dynamic range and response properties of the output cells, that is, the RGCs. Unbalanced synaptic interactions may also be the mechanistic explanation for the marked increase in spontaneous ganglion cell spike activity in transplanted retina [38].

## 6. Conclusions

The RP retina retains functional connections with the brain as shown originally by Drager and Hubel [16] who found that the 10 Hz rhythmic spike discharges they recorded from the optic track of *rd1* mice were also present in single unit recordings in the visual cortex. Thus it is likely that the increased level of spontaneous activity that has been described in animals models of RP is also be present in patients with degenerative retinal disease and may participate in the generation of the phantom visual images that are reported by some RP patients [39–41]. These sensations are not continuous, as one might expect they would be if produced by sustained rhythmic spike activity. They are described as being intermittent, as if produced by “lights” that twinkle, flash, or shimmer. This, however, does not rule out the possibility that spontaneous RGC spike activity is the underlying substrate for this phenomenon that when processed by normal or *rd1*-modified CNS circuitry gives rise to discontinuous visual sensations. In any case uncontrolled spontaneous spike activity would be expected to degrade the action potential encoded messages RGCs send to the brain and thus hinder attempts to restore vision using electrical or optical prosthetics designed to directly evoke RGC spike trains that the brain can interpret as meaningful visual information. 

Research designed to evaluate the treatment of RP using electronic or optical retinal prosthetics has not considered the influence that increased spontaneous RGC spike discharge might have on the successful use of prosthetics. While electrical stimulation of the retina in blind subjects can evoke the sensation of light and provide a rudimentary means of detecting motion, it has not been possible to use this approach to elicit the complex pattern percepts that are associated with more robust visual function [2]. Whether this has to do with the degradation of the retinal output signal by increased “noise” due to maintained rhythmic spike activity is not known but worthy of further investigation. Thus far studies focused on optical prosthetics have demonstrated that genetic incorporation of light-sensitive proteins, which included either melanopsin [5] or channelrhodopsin-2 alone [3, 4, 9] or coexpressed with halorhodopsin [7, 8], can restore light-evoked spike production in RGCs in animal models of RP; they have not been attempted in human subjects. Here again, however, the influence of increased spontaneous activity has not been addressed, but will need to be considered in order for treatments based on optical prosthetics to be optimized.
